# Emerging regulated cell death mechanisms in bone remodeling: decoding ferroptosis, cuproptosis, disulfidptosis, and PANoptosis as therapeutic targets for skeletal disorders

**DOI:** 10.1038/s41420-025-02633-3

**Published:** 2025-07-21

**Authors:** Hai-Ting Hu, Zhen-Yu Zhang, Zi -Xin Luo, Hui-Bo Ti, Jun-Jie Wu, Hao Nie, Zheng-Dong Yuan, Xian Wu, Ke-Yue Zhang, Shu-Wen Shi, Yi-Qing Qian, Xin-Chen Wang, Jing-Jing Wu, Xia Li, Feng-Lai Yuan

**Affiliations:** Institute of Integrated Chinese and Western Medicine, The Hospital Affiliated to Jiangnan University, Wuxi, Jiangsu 214041 China

**Keywords:** Apoptosis, Diseases

## Abstract

The adult skeleton preserves its structural and functional integrity through continuous bone remodeling, a process tightly regulated by osteoblasts, osteoclasts, and osteocytes. Disruptions to this balance contribute to skeletal pathologies like osteoporosis and periodontitis, underscoring the need to understand the mechanisms governing bone homeostasis. Regulated cell death (RCD) plays a key role in bone remodeling by modulating the activity of osteoblasts and osteoclasts. Recent advances have revealed novel RCD modalities: ferroptosis, cuproptosis, disulfidptosis, and PANoptosis, each with unique molecular mechanisms and pathophysiological implications in bone disorders. So we want to elucidate the molecular mechanisms, signaling cascades, and roles of these four novel RCD modalities in bone remodeling and skeletal homeostasis. We explore their potential involvement in bone-related pathologies, emphasizing the crucial roles of osteoblasts, osteoclasts, and osteocytes in maintaining skeletal integrity. By synthesizing emerging evidence, we aim to identify therapeutic targets and propose innovative strategies for managing skeletal disorders, advancing research in bone health and providing novel insights for clinical translation.

**Figure:**
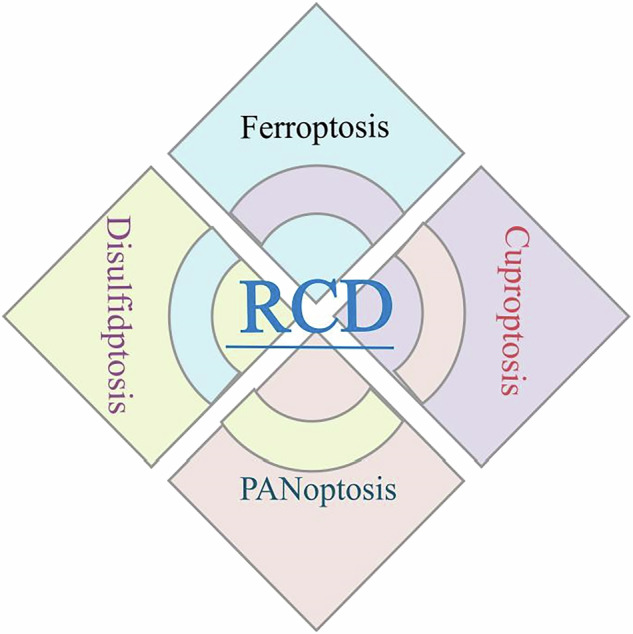
Emerging regulated cell death mechanisms in bone remodeling.

Emerging regulated cell death mechanisms in bone remodeling.

## Facts

- ACSL4-mediated lipid peroxidation may exacerbate osteoporosis, while GPX4 deficiency induces osteoblast death; iron chelators, however, may aid bone regeneration through Ferroptosis in Bone Metabolism.
- Osteoclasts (with high mitochondrial activity) may respond differently to FDX1/DLAT-dependent cuproptotic stimuli than osteoblasts, though the mechanism remains unclear.
- SLC7A11 overexpression promotes both cell death and survival, presenting a microenvironmental regulation dilemma about Disulfidptosis in Bone Metabolism.
- PANoptosome components exhibit disease-specific heterogeneity, raising debate over their unified mechanism.

## Open questions

- Whether we can combine targeting multiple RCDs to treat bone diseases, will this approach trigger compensatory death pathway activation?
- Does gut microbiota-derived iron and copper modulate bone death pathways?
- Are senescent osteocytes more prone to PANoptosis, driving age-related osteoporosis?
- Can we design dual-targeting drugs to inhibit PANoptosis while promoting osteogenesis?

## Introduction

The adult skeleton undergoes continuous bone remodeling, a vital process that maintains its structural robustness and functional integrity throughout life [1]. This process involves the replacement of old or damaged bone tissue, beginning with osteoclast-mediated resorption and concluding with osteoblast-driven bone formation in specialized compartments [2]. The equilibrium of bone remodeling is tightly regulated by osteoblasts, osteoclasts, and osteocytes, each playing distinct yet vital roles in bone metabolism [3]. Alterations in this homeostatic equilibrium may result in the pathogenesis of multiple osseous disorders, including osteoporosis, (OP) periodontal disease, and other pathological states associated with aberrant bone metabolism, underscoring the fundamental significance of regulated bone remodeling processes in maintaining skeletal integrity [4].

Recent studies have underscored the pivotal role of programmed cell death (PCD) in regulating tissue homeostasis, coordinating developmental processes, and controlling osteoclast and osteoblast activity, all of which are essential for bone remodeling and skeletal homeostasis [5]. PCD is conventionally classified into two distinct modalities: accidental cell death (ACD) and regulated cell death (RCD). ACD occurs as a consequence of unregulated cytotoxic insults, whereas RCD represents an evolutionarily conserved, molecularly orchestrated biological process that plays pivotal roles in morphogenesis, tissue equilibrium maintenance, and pathophysiological cascades [6].

Recent advances have identified several novel forms of RCD, each characterized by distinct molecular mechanisms and signaling pathways. Among these, ferroptosis, cuproptosis, disulfidptosis, and PANoptosis have attracted significant attention due to their potential involvement in various diseases, including bone disorders [7, 8]. Ferroptosis, an iron-dependent RCD, is initiated by lipid peroxide accumulation, causing membrane oxidation and cell death. This process contributes to skeletal pathologies through iron-mediated oxidative damage, disrupting bone remodeling via impaired osteoblast-osteoclast coupling [7, 9]. Cuproptosis, a copper-dependent RCD, arises from copper ion accumulation, which causes protein aggregation and mitochondrial dysfunction. The importance of copper homeostasis in bone metabolism is well-established, with disruptions potentially leading to bone abnormalities [7, 10]. Disulfidptosis represents a novel RCD modality mediated by pathological disulfide accumulation, particularly cystine, inducing intracellular redox imbalance. This cellular demise pathway is mechanistically associated with metabolic pathways involving SLC7A11, NADPH, and the Rac1-WAVE complex, suggesting a role in bone disorders associated with metabolic dysregulation [8]. Lastly, PANoptosis, an inflammatory form of PCD that incorporates features of pyroptosis, apoptosis, and necroptosis, is potentially triggered by inflammatory stimuli. Its involvement in bone diseases marked by chronic inflammation highlights its relevance in bone biology [11].

This review critically examines novel PCD modalities, exploring their molecular mechanisms, signaling pathways, and potential roles in bone remodeling. By deepening the understanding of these processes, the review seeks to uncover novel therapeutic targets for managing bone disorders and stimulate the development of innovative treatment strategies, thereby advancing the field of skeletal health research.

## Mechanistic insights into ferroptosis, cuproptosis, disulfidptosis, and PANoptosis pathways

The process of cell death within skeletal component cells is crucial for bone development, maintenance, repair, and overall functional integrity [12]. The Cell Death Nomenclature Committee classifies cell death into ACD and RCD according to their distinct functional features [13]. ACD refers to uncontrolled cellular death resulting from accidental or unintended injury, while RCD is governed by genetically encoded molecular machinery, playing essential roles in organismal development, tissue homeostasis, and disease pathogenesis. RCD can be pharmacologically or genetically manipulated to modulate its occurrence [14]. Comprehensive research on bone cells, especially osteoclasts and osteoblasts, along with related therapeutic strategies, has demonstrated that RCD-mediated cell death profoundly impacts the biochemical and cellular dynamics of the bone microenvironment. This influence plays a critical role in bone remodeling and the advancement of bone-related disorders [15, 16].

Conventional PCD mechanisms, such as necrosis, apoptosis, autophagy, and necroptosis, operate through unique molecular processes and signaling cascades, triggering bone cell death in response to external stimuli and internal disruptions [17]. However, during treatment, malignant bone cells may adopt various strategies to evade these RCD pathways, resulting in resistance to bone disease therapies and cellular resistance to death [18]. Consequently, the identification of novel RCD modalities capable of overcoming these defense mechanisms represents an innovative approach to combating bone disorders. In recent years, emerging forms of cell death have gained attention for their potential role in regulating bone metabolism. Ferroptosis and cuproptosis, both metal ion-dependent processes, are initiated by disruptions in iron and copper metabolic pathways, respectively [19]. Disulfide stress-induced disulfidptosis encompasses a complex interplay of cell death pathways, predominantly propelled by disulfide strain, and exhibits distinct mechanisms, traits, and biomarkers [20]. PANoptosis, a cell death mechanism that integrates various death pathways, is gaining more attention due to its role in bone-related disorders [21].

## Cuproptosis

Cuproptosis, a recently identified form of RCD dependent on copper ions, was first proposed by Tsvetkov et al. in 2022 [22]. The mechanism is akin to zinc- and iron-mediated cell death but differs from other forms of cell death, including apoptosis, necroptosis, and necrosis. Copper, a vital element in biological systems, serves as a cofactor in numerous enzymatic reactions and is involved in crucial processes such as oxidative stress defense, lipid processing, and energy generation [23].

### Intake, absorption, excretion of copper ions, and their regulation of bone metabolism

In biological systems, copper ions are primarily found in two states: the reduced form, cuprous ion (Cu^+^), and the oxidized form, cupric ion (Cu^2+^). The dietary Cu^2+^ is first converted to Cu^+^ by metalloreductases from the STEAP family, which are situated on the cell membrane surface [24]. Following this, the Cu^+^ is taken up by epithelial cells in the small intestine via the solute carrier family 31 member 1 (SLC31A1), also referred to as copper transporter 1 (CTR1). Upon uptake, about 95% of copper ions associate with plasma proteins and are circulated in the blood to different organs and tissues [25]. The liver is the main organ for maintaining copper balance in the diet, overseeing the disposal of any surplus via bile secretion. It is central in storing and expelling copper, processing excess amounts, integrating them into bile, and eliminating them via bile secretion, which is the main method for copper’s exit from the body [26]. ATPases Cu transporting alpha (ATP7A) and beta (ATP7B) are crucial in copper uptake and efflux [27]. When copper levels in the periphery decline, ATP7A releases copper from liver stores, delivering it to the bloodstream to ensure adequate concentrations in peripheral tissues [28]. ATP7A guides copper to the trans-Golgi network and vesicles within cells, regulating its subcellular distribution and assisting in copper’s integration into specific proteins. Conversely, ATP7B manages the export of intracellular copper. In situations of copper overload, cytoplasmic Cu^+^ in hepatocytes links with metallothionein 1 (MT1), which then transports it to the bile canaliculi membrane, facilitating the body’s removal of excess copper [29].

Through CRISPR/Cas9-based whole-genome loss-of-function screens, key metabolic pathways controlling cell death due to Cu ion carrier-mediated mitochondrial respiration were uncovered [30]. Seven genes vital for cuproptosis—a condition marked by the buildup of Cu^2+^ within mitochondria, resulting in protein aggregation, Fe-S cluster disruption, and subsequent necrosis—were identified. These genes encompass ferredoxin 1 (FDX1) and lipoyltransferase 1 (LIPT1) from the lipoic acid pathway, as well as lipoic acid synthase (LIAS), dihydrolipoamide dehydrogenase (DLD), dihydrolipoamide S-acetyltransferase (DLAT), pyruvate dehydrogenase E1 subunit alpha 1 (PDHA1), and pyruvate dehydrogenase E1 subunit beta (PDHB) [22, 31, 32]. Despite being independent of the electron transport chain (ETC), cuproptosis impacts mitochondrial respiration. FDX1 encodes a small iron-sulfur cluster protein that plays a crucial role in mitochondrial cytochrome reduction and steroid hormone biosynthesis [33]. Pioneering work by Tsvetkov et al. [22] demonstrated that copper overload selectively disrupts mitochondrial lipoylation metabolism, particularly targeting DLAT, thereby impairing cellular viability. Their studies revealed FDX1’s dual functions in cuproptosis: serving as a reductase converting Cu^2+^ to the more toxic Cu⁺, and acting as an essential upstream regulator of lipoic acid synthesis and protein lipoylation [22, 34]. Genetic ablation of FDX1 completely abrogates cuproptosis, establishing its central regulatory role in this pathway, where DLAT and heat shock protein 70 (HSP70) function as downstream effectors. Subsequent investigations uncovered novel regulatory mechanisms involving copper-mediated SEC14L3 upregulation, which suppresses ERK/YY1 axis activity to enhance FDX1 expression. This cascade ultimately promotes lipoylated-DLAT (lip-DLAT) oligomerization and induces cuproptosis, thereby inhibiting hepatocellular carcinoma progression [35]. Complementary studies identified chlorophyllin as an FDX1-interacting compound that facilitates DLAT oligomerization and oxidative stress in pancreatic cancer cells [36, 37]. Additionally, database screenings have identified further genes linked to cuproptosis, including both positively and negatively regulated genes, as well as copper transporters such as SLC31A1, ATP7A, and ATP7B [22]. Further protein-based studies are necessary to confirm the links between transcript alterations, associated targets, and the end-products of downstream enzymatic reactions.

The interaction between Cu^2+^ ions and lipoylated DLAT, followed by oligomerization, may have significant implications in both physiological and pathological contexts [22, 38]. This interaction and subsequent oligomerization could affect the efficiency and regulation of the tricarboxylic acid (TCA) cycle, thereby influencing cellular energy metabolism [39]. Moreover, Cu^2+^ binding to lipoylated enzymes within the TCA cycle, particularly DLAT, can induce abnormal aggregation of DLAT, leading to the formation of insoluble protein aggregates [40]. This aggregation, triggered by Cu^2+^ ions, results in the loss of protein functionality and ultimately contributes to cuproptosis. Proteins containing Fe-S clusters, which serve as essential cofactors for enzymes in the ETC and various biochemical reactions, are pivotal in this scenario [22, 23]. Elesclomol (ES), a Cu^2+^ binder, forms ES-Cu complexes and facilitates Cu^2+^ entry into cells [41]. Ferredoxin 1 (FDX1, an Fe-S cluster protein) then reduces Cu^2+^ to Cu^+^. Notably, glutathione (GSH) can chelate intracellular Cu^+^, reducing free Cu^+^ levels and mitigating the damage caused by ES-Cu complexes [42]. Inhibition of GSH production, however, leads to Cu^+^ accumulation. Significantly, FDX1 cannot be oxidized by ES or Cu^2+^ ions individually, which further underscores the importance of the Cu^2+^ complex in the anticancer effects of ES and its derivatives.

Upon entering cells, copper ions bind to chaperone proteins and are directed to diverse cellular locations where they modulate a range of functions [43]. In the cytoplasm, the copper chaperone for superoxide dismutase (CCS) guides copper ions to targeted proteins, including superoxide dismutase 1 (SOD1), which needs copper at its active center. SOD1 converts superoxide radicals (O2-) into oxygen (O^2^) and hydrogen peroxide (H_2_O_2_), thereby safeguarding cells against oxidative damage [44]. Copper is also a crucial coenzyme in energy generation through mitochondrial respiration [45, 46]. The entry of copper into mitochondria is primarily facilitated by the cytochrome c oxidase copper chaperone COX17, which shuttles Cu(I) from the cytoplasm to the inner mitochondrial membrane. Inside the mitochondria, copper contributes to the synthesis of cytochrome c oxidase subunits 1 (SCO1) and SCO2, aiding in the assembly of cytochrome c oxidase subunit 2 (MT-CO2, also referred to as COX2)[47, 48]. The assembly proteins COX16 and cytochrome c oxidase assembly factor 6 (COA6) then help in transferring copper from SCO1 and SCO2 to MT-CO2[49]. Copper ions are conveyed to the trans-Golgi network via the copper chaperone for antioxidant 1 (ATOX1), which binds to Cu(I) in the cytoplasm and aids in its transport to copper-transporting ATPases like ATP7A and ATP7B within the Golgi complex [50, 51]. CCS and ATOX1 both facilitate the transport of copper into the nucleus, where copper ions are involved in the activation of various transcription factors [52]. The human body usually contains copper in the range of 100 to 200 milligrams (mg) [53]. Elevated copper levels are frequently detected in the cancerous tissues and/or blood of patients with different types of cancers, such as breast, lung, gastrointestinal, oral, thyroid, gallbladder, cervical, ovarian, kidney, and prostate cancers. Within cells, free and labile copper ions can trigger Fenton-like reactions, producing reactive oxygen species (ROS) that damage iron-sulfur clusters [54, 55]. Proteins like metallothioneins and GSH are crucial for reducing the accumulation of Cu(I) by binding and sequestering excess copper ions, thereby protecting the cell from toxicity [56, 57]. While copper is vital for tumor development and progression, the molecular mechanisms underlying copper-induced toxicity and subsequent cell death remain incompletely understood [53, 58, 59].

The development of bone-related abnormalities associated with copper dysregulation likely involves several mechanisms. Bone cells require higher copper concentrations than most other tissues to maintain metabolic activities [60, 61]. Approximately two-thirds of the total copper in the human body is found in muscles and bones [53, 62]. Numerous studies have established copper’s essential role in regulating bone metabolism. Copper impacts the synthesis of bone collagen and the metabolism of the bone matrix, both of which are vital for proper bone development [63, 64]. Additionally, copper helps increase bone density and strength, which are crucial for bone growth and overall integrity. Copper also influences collagen accumulation during the differentiation of mesenchymal stem cells into osteoblasts, thereby affecting bone formation at the molecular level [65, 66]. Copper ions regulate bone metabolism by interacting with various cellular signaling pathways, such as the hypoxia-inducible factor (HIF)-1 pathway, ERK1/2 signaling, integrin signaling, and Toll-like receptor (TLR) signaling pathways [67, 68].

**Fig. 1:**
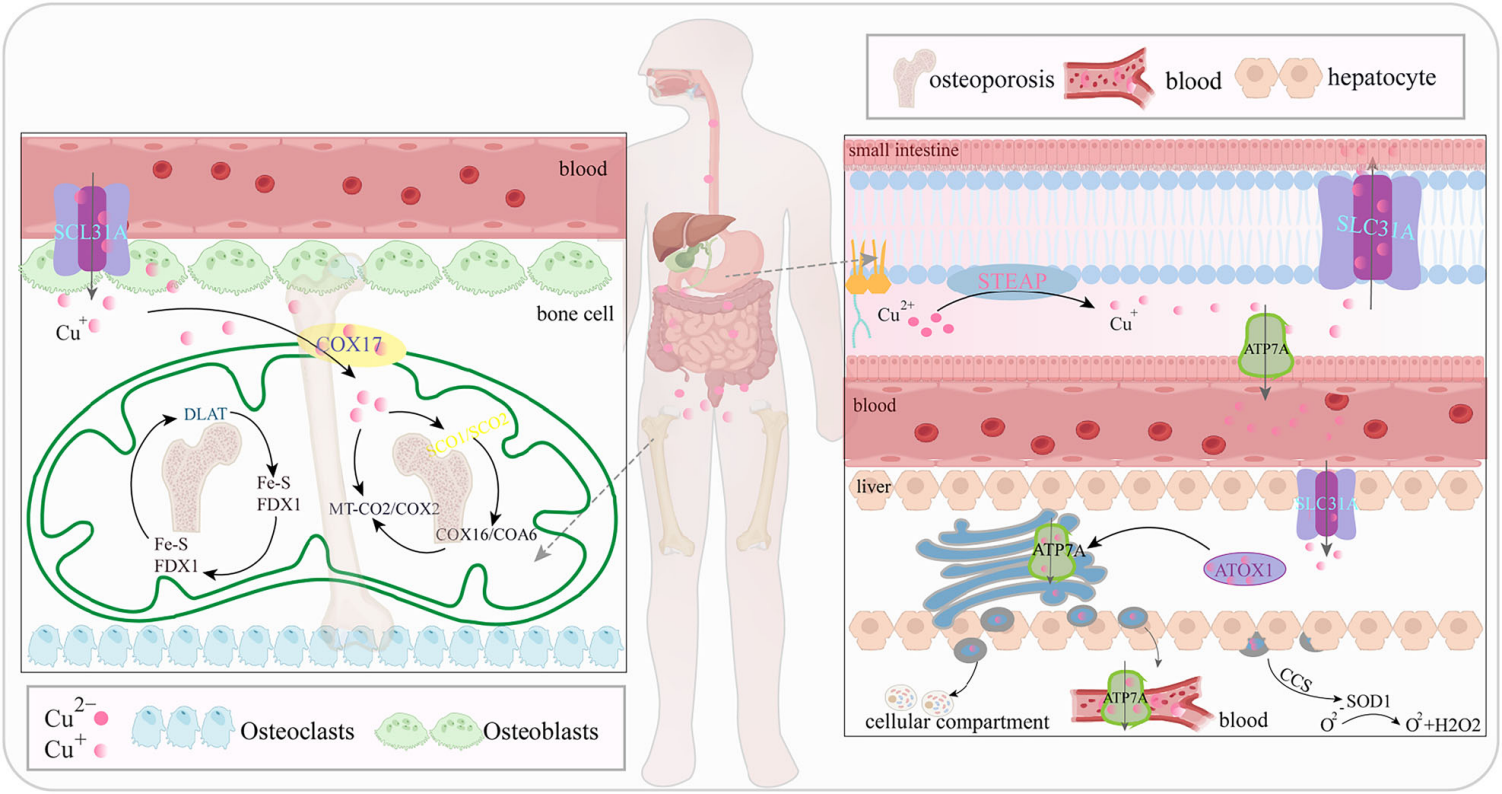
Intake, absorption, excretion of copper ions, and their regulation of bone metabolism. The dietary Cu^2+^ is first converted to Cu^+^ by metalloreductases from the STEAP family. Following, the Cu^+^ is taken up by epithelial cells in the small intestine via the SLC31A1, also referred to as CTR1. ATP7A delivers copper to the bloodstream and guides it to the trans-Golgi network and vesicles.ATP7B manages the export of intracellular copper. In the cytoplasm, the copper chaperone for CCS guides copper ions to SOD1. SOD1 converts O^2-^ into oxygen O^2^ and H_2_O_2_. FDX1 serves as a reductase converting Cu^2+^ to the more toxic Cu^+^, and acts as an essential upstream regulator of lipoic acid synthesis and protein lipoylation. COX17 facilitates copper into mitochondria. Inside the mitochondria, copper contributes to the synthesis of SCO1 and SCO2, aiding in the assembly of MT-CO2, also referred to as COX2.

Imbalances in copper homeostasis, such as excessive accumulation or defective transport, can cause intracellular copper levels to surpass the limits established by homeostatic controls. This can result in cellular toxicity and adversely affect bone health by influencing osteoblasts and osteoclasts [69, 70]. Extensive research has demonstrated that copper deficiency exerts detrimental effects on both osteoblastic and osteoclastic functions. Multiple studies using copper-deficient murine models have consistently reported significant reductions in the population of both osteoblasts and osteoclasts within bone tissue [65, 71–73]. This dual cellular depletion disrupts the delicate equilibrium between bone formation and resorption, leading to profound consequences for skeletal homeostasis [64, 74]. The impaired bone remodeling process manifests as: (1) diminished bone mass and compromised mechanical strength; (2) arrested bone formation and growth retardation; (3) reduced mineral deposition and matrix maturation; and (4) delayed ossification of growth centers. These pathological changes collectively underscore the essential role of copper in maintaining proper bone cell dynamics and skeletal integrity. Moreover, changes in copperconcentrations can disrupt proteasome function, causing a buildup of misfolded proteins and exacerbating bone abnormalities.These findings highlight copper’s role in regulating various aspects of bone remodeling, including osteoblast and osteoclast function, bone matrix formation and turnover, bone density and strength, and metabolic processes within bone tissue (Fig. 1).

### Cuproptosis in bone remodeling

Cuproptosis, a unique type of cellular demise caused by the buildup of intracellular copper ions, is attracting growing interest as a potential therapeutic target in various illnesses. Notably, the impact of copper on bone health has become a significant research focus lately. A cluster of genes known as copper ion-induced cell death-related genes (CRGs), which includes DLAT, MTF1, GLS, CDKN2A, PDHA1, PDHB, and DLD, have been pinpointed as important modulators of bone metabolism, influencing the activities of osteoblasts and osteoclasts [75].

GLS plays a key role in the regulation of osteogenesis and adipogenesis in skeletal stem cells (SSCs) by modulating cell fate and bone development, which it does by affecting histone acetylation and glutamine metabolism [76]. The P16 (INK4A) protein, produced by CDKN2A, is a biomarker for cellular aging. Its overexpression hampers osteoblast mineralization and concurrently boosts the number of osteoclasts, thereby disturbing the balance of bone homeostasis [77].

Recent research has also underscored the influence of CRGs on the differentiation of osteoblasts and osteoclasts. For instance, inhibition of PDHA1 may promote osteoblast proliferation, while its activation can suppress inflammatory factor release from osteoblasts [78]. PDHB, through its interaction with NIMA-associated kinase ten, influences osteoclast differentiation and maintains mitochondrial homeostasis, thereby regulating osteoblast differentiation [79]. DLD modulates glycolysis and affects osteoclast differentiation, further underscoring the importance of metabolic pathways in bone cell function [79, 80].

While the exact mechanisms through which CRGs influence bone biology remain incompletely understood, previous investigations have demonstrated that several of these genes directly or indirectly regulate bone formation and resorption. Current investigations into these genes are promising for enhancing our grasp of bone biology and revealing novel therapeutic approaches for bone-related ailments.

Copper ions, especially those implicated in cuproptosis, are pivotal in bone metabolism, facilitating the functions of osteoblasts and osteoclasts.About two-thirds of the body’s copper is stored in muscles and skeletal structures,which is essential for proper bone development, enhanced bone density, and improved bone strength [81]. By modulating collagen production and bone matrix metabolism, copper ions facilitate bone formation and growth, highlighting their vital role in skeletal health [82].

On a cellular and molecular scale, copper ions interact with various signaling pathways, such as the HIF-1 pathway, ERK1/2 signaling, integrin signaling, and TLR signaling, significantly impacting bone metabolism. Disruptions in copper homeostasis, whether due to excess accumulation or improper transport, can induce toxic effects on cells and undermine bone health [83].

Copper deficiency primarily affects osteoblasts, causing them to suffer from functional deficits, which in turn lead to decreased activity, impaired bone turnover, lower bone density and resilience, and altered bone development and growth. It also results in reduced mineralization and compromised ossification at growth zones. Research on copper-depleted animals indicates a decrease in the osteoblast and osteoclast populations in bone tissue, yet the functionality and viability of mature osteoclasts are not compromised. This is linked to SOD1 deficiency, which prompts the generation of ROS, thereby disrupting the redox equilibrium in osteoblasts and leading to compromised survival and osteoblast differentiation [84].

Conversely, elevated copper levels negatively affect both osteoblast and osteoclast functions. High copper concentrations inhibit the differentiation of MSCs into osteoblasts by stabilizing HIF-1α and downregulating Runx2, both of which are essential for bone formation [85]. Additionally, copper directly impairs osteoclast resorption activity, with a notable reduction in the number of mature osteoclasts at high concentrations [86]. This toxicity emphasizes the critical need for precise copper regulation to maintain optimal bone health, as imbalances can induce copper-mediated cell death, leading to detrimental effects on bone structure and function [70].

Copper-doped biomaterials, such as bioactive glasses and hydrogels, have demonstrated promise in enhancing bone repair and regeneration, both by promoting osteoblast function and providing antibacterial properties [87].

However, no clear scientific reports exist on the regulation of cuproptosis in osteocytes, indicating that this area of research is still in its infancy and holds potential for future breakthroughs. The regulation of cuproptosis in osteocytes likely involves a complex interplay of copper homeostasis, mitochondrial function, redox regulation, and multiple signaling pathways, necessitating further investigation to inform the development of targeted therapies for bone remodeling.

### Detection of cuproptosis

#### Detection of cuproptosis based on cu content

Research has demonstrated that Elesclomol, at concentrations as low as 40 nM, can significantly elevate intracellular copper levels (as measured by inductively coupled mass-spectrometry [ICP-MS]) and trigger cell death within 24 hours [22, 88]. Pre-treatment with copper chelators or the absence of copper ions can reverse these effects, thus confirming that cell death is caused by copper ion accumulation rather than the Cu ionophore itself [30, 89]. Therefore, during cuproptosis, a marked increase in intracellular copper levels is expected, necessitating quantification whenever cell death is observed under copper stress [90, 91].

Although ICP-MS provides high precision and sensitivity for quantifying metal ions, its complexity and equipment requirements limit its widespread application [91, 92]. Alternative methods, such as colorimetric assays, fluorescent probes, and electrochemical techniques, are needed to rapidly and accurately measure copper in serum and cell lysates.

#### Colorimetric method

Colorimetric assays produce distinct color complexes with copper ions, where the intensity of the color is directly proportional to the copper concentration. These assays are straightforward, highly sensitive, precise, and well-suited for high-throughput screening, as they can be easily measured using a microplate reader [93].However, they may be affected by interference from metal chelators and necessitate the use of a microplate reader [91]. Representative examples of such kits are available from companies like MesGen, Elabscience, and BioAssay Systems [94, 95].

#### Fluorescent probe method

Fluorescent probes, known for their high sensitivity and rapid response, are extensively employed in sensing and imaging applications. The fluorescence of noble metal nanoclusters and quantum dots is quenched by copper ions, rendering these probes extremely effective for detecting copper [91]. These probes offer increased sensitivity, accuracy, and throughput, though they necessitate the use of a fluorescent reader [96, 97].

#### Electrochemical method

The electrochemical method uses highly conductive materials, such as Ti3C2Tx/MWNTs-Au, as working electrodes. Binding of Cu ions results in measurable changes in the electrical signa [98]. However, this method may experience high background signals and non-visual results. Combining electrochemistry with electrochromic materials, like CdS QDs/WO3 nanoflakes, enables visual detection of Cu ions through color changes, allowing for semi-quantitative field detection [99]. This approach requires an electrochemical workstation.

#### Detection of cuproptosis based on morphology

The ability to restore cell death effects through copper chelators, combined with the presence of copper deposition, suggests that cuproptosis is occurring [100, 101]. Morphological evaluations can distinguish cuproptosis by observing changes in cell membranes and organelles. For instance, Yang et al. have highlighted the characteristic morphological hallmarks of cuproptosis, such as cell membrane rupture, mitochondrial contraction, endoplasmic reticulum (ER) damage, and chromatin fragmentation. These features can be visualized using transmission electron microscopy (TEM) [90]. Although morphological analysis provides a clear visual indication, it requires intricate sample preparation and can be subject to personal bias. As a result, it is typically used in conjunction with quantitative methods to provide initial validation of cuproptosis.

#### Detection of cuproptosis based on molecular biology

Cuproptosis, a newly discovered type of cell death, is closely associated with disturbances in copper homeostasis and mitochondrial respiration, often triggered by copper ionophores [102]. This process entails distinct changes in the levels of proteins related to cuproptosis, such as those involved in lipoylation, the ETC, and heat shock protein 70 (HSP70) [103]. Additionally, disturbances in Cu transport and Cu chaperones can further disrupt Cu homeostasis and exacerbate a range of diseases, including genetic, neurodegenerative, cardiovascular, and oncological conditions [23, 104–107].

In cuproptosis, the FDX1 can be measured using techniques like qRT-PCR, western blot, immunohistochemistry (IHC), and ELISA [108]. DLAT can be detected by immunofluorescence, gel electrophoresis, high-performance liquid chromatography (HPLC), and other methods, and acts as a marker for cuproptosis [109]. The lipoylation of DLAT, essential for the pyruvate dehydrogenase (PDH) complex and the TCA cycle, is regulated by FDX1 in collaboration with LIAS [22].

Proteins that play a key role in Fe-S cluster assembly and transfer include NFS1-ISD11, frataxin, ferredoxin, ferredoxin reductase, Mrs3/4, Isa1/2, Iba57, Ind1, Nfu1, and Fe-S subunits like NDUFS1, NDUFV1, SDHA, and UQCRFS1 [110–112]. During cuproptosis, these proteins can be assessed using techniques such as western blot, IHC, and quantitative real-time PCR (qRT-PCR) [113].

HSP70 can be measured by Western blot, IHC, and qRT-PCR. HSP70 plays a protective role by preventing protein misfolding, aggregation, and disruption of complex assembly under stress conditions. [114].

In conclusion, the molecular biology of cuproptosis is characterized by complex interactions between Cu homeostasis, mitochondrial respiration, and the expression of specific proteins. Thorough evaluation of these interactions is essential for understanding and confirming the occurrence of cuproptosis.

## Ferroptosis

### The mechanism of ferroptosis in osteocytes

First identified by Dixon et al. [115] in 2012, ferroptosis is an iron-dependent form of rRCD that occurs due to the buildup of lipid ROS inside cells. This accumulation leads to peroxidation-induced damage to the cell membrane. Ferroptosis involves lipid peroxidation and subsequent cell death, which is mediated by ferrous iron or lipoxygenase. It is essential for regulating various physiological processes. The core mechanisms of ferroptosis are closely linked to iron and lipid metabolism, the cystine–glutamate antiporter (system Xc-)/GSH/glutathione peroxidase 4 (GPX4) pathway, and TP53 gene regulation [116–119]. Key organelles involved in this process include mitochondria, the ER, and lysosomes [120–122]. Additionally, miRNAs regulate ferroptosis-related factors under different pathophysiological conditions [123, 124].

The process of ferroptosis is mainly triggered by the buildup of iron, with proteins that govern iron homeostasis being key in regulating this phenomenon. In particular, ferrous iron (Fe^2+^) is a crucial catalyst for lipid peroxidation and ferroptosis in bone cells [115]. Proteins like transferrin (TF), heme oxygenase 1 (HO-1), and the transferrin receptor (TFRC) are instrumental in controlling intracellular iron levels, thereby impacting the occurrence of ferroptosis [125–127]. Elevated cellular Fe (II) levels promote lipid peroxide accumulation through iron-dependent Fenton reactions that generate ROS, and by activating iron-containing enzymes such as lipoxygenase (LOX) and cytochrome P450 oxidoreductase (POR), which further drive lipid peroxidation and ferroptosis [103, 118, 128]. The inhibition of ferroptosis by iron chelators, which reduce iron levels, underscores the critical role of excess iron in this process [129, 130].

### Lipid peroxidation in bone cells

In the context of bone metabolism, polyunsaturated fatty acids (PUFAs) are highly prone to peroxidation triggered by ROS [131]. When phospholipids with PUFA chains experience oxidative stress, they form phospholipid hydroperoxides (PLOOHs), which can disrupt membrane stability unless GPX4 intervenes to reduce them [131, 132]. Enzymes such as LPCAT3 and ACSL4 play a crucial role in activating PUFAs and integrating them into phospholipids, thereby facilitating ferroptosis [133]. Additionally, the site-specific oxidation of PUFAs, catalyzed by LOX, further promotes ferroptosis [134].

### The system Xc-/GSH/GPX4 pathway in bone cells

The system Xc-, consisting of SLC7A11 and SLC3A2, enables the transport of cysteine in exchange for glutamate [135]. When extracellular glutamate levels rise, this system is inhibited, leading to ferroptosis, potentially explaining the toxic effects of glutamate in bone tissue. GSH, derived from cysteine and glutamate, is vital for Fe-S cluster assembly [126]. GPX4, a major ferroptosis inhibitor, detoxifies PLOOHs by reducing them to harmless alcohols, thus preventing lipid peroxidation [136]. Cells that synthesize cysteine from methionine via the transsulfuration pathway show enhanced resistance to ferroptosis induced by system Xc- blockade [137].

### TP53 regulation of ferroptosis in bone cells

TP53 exerts a dual regulatory role in ferroptosis within bone cells. Upon activation, it downregulates SLC7A11, impairing cysteine uptake and GSH synthesis, thereby promoting ferroptosis [135, 138]. Conversely, TP53 inhibits ROS production through interaction with dipeptidyl peptidase 4 (DPP4), leading to CDKN1A induction, which enhances GSH synthesis and elevates intracellular NADP^+^ and GSH levels [135, 138, 139].

Additionally, TP53 modulates spermine/spermidine N1-acetyltransferase 1 (SAT1) [140], facilitating arachidonic acid oxidation and lipid peroxide generation [141–143]. Variants of p53 exhibit diverse effects on ferroptosis; for example, dexamethasone-induced ferroptosis in osteoblast-like MC3T3-E1 cells is regulated by p53. Knockdown of p53 expression via small interfering RNA (siRNA) reverses the suppression of SLC7A11 and GPX4, thereby reducing ferroptosis [144, 145]. These results underscore the involvement of TP53 in regulating ferroptosis and imply that targeting ferroptosis suppression could serve as a potential therapeutic approach for managing osteonecrosis of the femoral head.

### Ferroptosis in bone remodeling

Bone remodeling is a dynamic process that involves both resorption and deposition phases, driven primarily by osteoclasts and osteoblasts [146–148]. Osteoclasts break down bone, thus releasing minerals and nutrients into the circulatory system. Concurrently, osteoblasts contribute to the formation of fresh bone, thereby preserving the skeletal structure’s stability and robustness [146, 149, 150]. Additionally, osteocytes, the most abundant cells in mature bone, play a critical role in regulating bone turnover. Complex signaling networks regulate osteocytes to sustain equilibrium between bone creation and breakdown, thereby maintaining optimal bone homeostasis [151–153].

#### In osteoblasts

Osteoblasts, which are key in bone construction, are susceptible to ferroptosis due to iron buildup [154]. Excess iron within osteoblasts initiates a sequence of reactions, predominantly via the Fenton process, leading to a rise in ROS production. These ROS not only damage cellular components but also disrupt key signaling pathways involved in osteoblast differentiation and function. Studies by Xia et al. and others have shown that iron-induced ROS inhibit the PI3K/Akt pathway, activating glycogen synthase kinase 3β (GSK-3β), which suppresses Runx2, a key transcription factor for osteoblast differentiation [155–157]. This suppression diminishes bone formation and mineralization. Tian Q et al. identified the RIPK1/RIPK3/MLKL pathway as essential for iron-induced osteoblast necrosis [158]. They discovered that RIPK3 substrates PGAM5 and DRP1 play key roles in mitochondrial homeostasis. Activation of RIPK3 leads to the recruitment of PGAM5 to the mitochondrial membrane, which in turn activates DRP1 and induces changes in mitochondrial permeability, culminating in osteoblast apoptosis. Ferroptosis in osteoblasts is marked by mitochondrial impairment and the discharge of damage-associated molecular patterns (DAMPs), which intensifies inflammation and oxidative stress within the bone microenvironment [159]. These changes collectively hinder osteoblast function, fostering bone resorption and disrupting bone turnover. Osteoblast precursors, originating from bone marrow stromal cells (BMSCs), migrate to areas of active bone remodeling, where they differentiate into osteoblasts tasked with the creation of new bone [160–163]. Ferroptosis in these precursor cells can disrupt this process, leading to bone remodeling dysregulation [164–167]. Rozen reported that ammonium ferric citrate dose-dependently inhibited BMSC activity and osteoblast differentiation, reduced bone nodule formation and calcium deposition, and increased ROS and lipid peroxide levels. Iron accumulation suppressed the Wnt signaling pathway, decreasing osteoblast differentiation, which could be reversed by Wnt activation. Zhang et al. demonstrated that radiation reduced hepcidin, increasing iron levels and inhibiting BMSC osteoblast differentiation through Runx2 reduction [168]. Han et al. found that ferrous citrate did not affect BMSC proliferation but increased ROS, inhibited c-Maf and Runx2 expression, promoted PPARγ, reduced calcium nodule formation, and increased lipid droplets. These effects were reversed by antioxidants. Conversely, Yao et al. reported that ferric ammonium citrate increased caspase-3 and BAX expression, decreased Bcl-2, and inhibited BMSC activity and proliferation, suggesting that iron accumulation not only disrupts osteoblast differentiation but also impacts BMSC viability [169].

#### In osteoclasts

Osteoclasts, the primary cells involved in bone resorption, play a crucial role in bone remodeling and the maintenance of bone balance [170]. Recent research has underscored ferroptosis, an iron-mediated form of controlled cell death marked by lipid oxidation, as a pivotal factor in governing osteoclast differentiation and their functionality. In a study by Zhong and colleagues, it was shown that osteoclast precursors (pre-OCs) are more susceptible to the TXNRD1 (thioredoxin reductase 1) inhibitor compared to bone marrow-derived monocytes during the in vitro differentiation of osteoclasts triggered by RANKL. The nuclear factor of activated T-cells 1 (NFATc1) emerged as a crucial player in this mechanism, as it increased the expression of SLC7A11 in pre-OCs, thereby augmenting their vulnerability to TXNRD1 inhibition [20]. In normal oxygen levels, the iron-starvation response and ferritinophagy, the autophagic breakdown of ferritin to liberate iron, are vital for osteoclast maturation. This is supported by evidence indicating that the suppression of HIF-1α, a pivotal controller of iron handling, curtailed these mechanisms and alleviated bone depletion in ovariectomized (OVX) osteoporotic rodents. The evidence was further supported by studies revealing that the selective blocking of HIF-1α, a principal modulator of iron homeostasis, decreased these activities and lessened bone loss in mice with OP triggered by OVX [20]. These results underscore the importance of iron availability and ferritinophagy in osteoclastogenesis. Furthermore, recent studies indicate that overexpression of EZH2 enhances osteoclast differentiation, a process vital for bone resorption, while EZH2 knockdown inhibits osteoclast differentiation, highlighting the delicate balance EZH2 maintains in regulating osteoclast activity [171].

#### In osteocytes

**Fig. 2:**
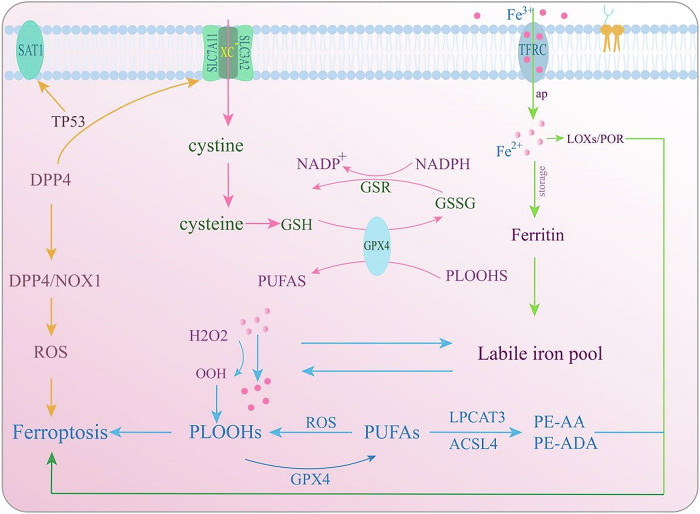
The mechanism of ferroptosis in osteocytes. The system Xc enables the transport of cysteine in exchange for glutamate. GPX4 detoxifies PLOOHs by reducing them to harmless alcohols, thus preventing lipid peroxidation. TP53 downregulates SLC7A11, impairing cysteine uptake and GSH synthesis, thereby promoting ferroptosis. Conversely, TP53 inhibits ROS production through interaction with DPP4, leading to CDKN1A induction, which enhances GSH synthesis and elevates intracellular NADP^+^ and GSH levels.

Ferroptosis, marked by iron-mediated lipid oxidation and membrane injury, has a substantial impact on the fate of osteocytes [165]. In diabetic scenarios, like diabetes-induced osteoporosis (DOP) in rodents, osteoblast ferroptosis results in a significant decrease in femoral bone density. This phenomenon is propelled by the upregulation of heme oxygenase-1 (HO-1), which is controlled by the NRF2 and c-JUN transcription factors [172]. Estrogen withdrawal also triggers iron accumulation in osteocytes, promoting ferroptosis and consequently reducing bone mineral density and increasing bone fragility [173]. The role of ferroptosis in bone loss has been further validated in various models, including osteocyte/osteoclast coculture systems and GPX4 knockout OVX mice, highlighting the contribution of ferroptotic osteocytes to osteoclastogenesis and exacerbated bone resorption [174, 175]. Ferroptosis regulation in osteocytes involves complex signaling pathways and gene expression alterations. The Nrf2 signaling cascade is central in regulating the expression of nuclear factor kappa-B ligand (RANKL) by modulating DNA methylation, a process influenced by DNA methyltransferase 3a (Dnmt3a) [165]. This mechanism is crucial for the induction of osteoclastogenesis via osteocytic ferroptosis. Moreover, the activating transcription factor 3 (ATF3) has been pinpointed as a pivotal factor in osteocytic ferroptosis. Its enhanced expression facilitates iron uptake and suppresses antioxidant defenses, fostering a conducive setting for ferroptosis [176]. ATF3’s dual function underscores the complex interplay between iron metabolism and antioxidant protection within osteocytes (Fig. 2).

### Detection of ferroptosis

Ferroptosis detection involves multiple approaches due to its distinctive cellular morphology and complex metabolic processes. Although specific assays for ferroptosis remain limited, several widely used methods are available.

#### Cellular state detection in ferroptosis

Researchers commonly use reagents such as MTT, CCK-8, PI, LDH, and ATP kits to assess cell viability and changes in cellular states during ferroptosis, based on the principle that ferroptosis induces alterations in cell viability and state [177].

MTT and CCK-8 assays are widely employed colorimetric methods for assessing cell viability. MTT is reduced to a blue-purple formazan compound by metabolically active cells, while CCK-8 generates a water-soluble product, making it applicable to both adherent and suspended cells [178]. However, MTT may not be suitable for suspended cells due to potential formazan loss, whereas CCK-8, though more costly, can yield false results due to its light red color, which may interfere with the phenol red in the culture medium [179, 180]. PI staining, used with flow cytometry or fluorescence microscopy, identifies dead cells with compromised membranes by emitting fluorescence [181]. LDH, a stable cytoplasmic enzyme, is released into the supernatant upon membrane damage, indicating cell death [182]. LDH, a stable cytoplasmic enzyme, is released upon membrane damage, indicating cell death. LDH assays quantify the formazan produced, which correlates with the number of dead or damaged cells [183]. ATP content measurement is another indicator of cell activity and viability, as ATP is rapidly depleted upon cell death [184]. Additional techniques, such as Hoechst and SYTOX Green dual staining, assess cell death by differentially labeling live and dead cells with distinct fluorescence [144, 145, 185]. PI staining and LDH assays are particularly useful for quantifying cell death, while ATP measurements provide insights into cellular activity.

#### Detection of subcellular organelles during ferroptosis

Ferroptosis involves the accumulation of lipid peroxides, which affect subcellular membranes and organelles. Monitoring changes in these organelles is crucial to understanding ferroptosis [115].

Mitochondria are pivotal in ferroptosis, contributing to ROS generation and iron regulation [186]. TEM can detect structural alterations in mitochondria, including swelling and cristae reduction [115]. For confocal microscopy, Mito-Tracker and MitoSox Red are used to specifically target mitochondria and mitochondrial ROS, respectively [186]. Additionally, flow cytometry enables the quantification of mitochondrial ROS levels, while probes like DIOC6, MitoTracker Deep Red FM, and TMRM assess changes in mitochondrial membrane potential and permeability during ferroptosis [186–188].

The ER undergoes increased viscosity, lipid peroxidation, and stress in response to ferroptosis inducers, leading to a reduction in monounsaturated fatty acids [189]. ER viscosity can be measured using two-photon phosphorescence lifetime imaging and ER-targeted fluorescent probes such as PV1. ER stress is further assessed by detecting proteins such as ATF4 and eIF2α via Western blot [189, 190].

Lysosomes are involved in iron metabolism, lipid peroxidation, and autophagy during ferroptosis [191]. Elevated iron or nitric oxide (NO) levels in lysosomes contribute to ferroptosis. Lysosomal cathepsin B (CTSB), a ferroptosis executor, can be detected by western blot, while qRT-PCR or Western blot can be used to assess genes and proteins related to ferroptosis via lysosomal pathways [192–194].

Lipid peroxide accumulation during ferroptosis also affects Golgi apparatus function, although the underlying mechanisms remain unclear [195]. Compounds that disperse the Golgi apparatus have been found to induce ferroptosis [196]. Golgi morphology can be analyzed using immunofluorescence staining with GM130 and fluorescence microscopy, revealing reduced Golgi dispersion during ferroptosis [196].

Mitochondria play a pivotal role in ferroptosis, with the detection of mitochondrial changes being a research priority. TEM is commonly used to observe morphological alterations or changes in membrane potential [197]. However, TEM has limitations, such as potential alterations in membrane structure during sectioning and interference from other double-membrane organelles, which hinder quantitative analysis [198].

#### Detection of genes, proteins, and transcription factors in ferroptosis

The regulation of ferroptosis relies on the interplay of numerous genes, transcription factors, and proteins. To analyze these components, researchers commonly use qRT-PCR and Western blot. qRT-PCR quantifies gene expression through fluorophore-based detection, while Western blot identifies protein levels via antibody binding [199].

The primary genes and proteins linked to ferroptosis play significant roles in regulating lipid metabolism, iron balance, and antioxidant defense mechanisms.ACSL4 and LPCAT3 promote ferroptosis by facilitating fatty acid activation and peroxidation, whereas ACSL3 and SCD1 reduce cancer cell sensitivity to ferroptosis. Although PTGS2 expression increases during ferroptosis, it is not considered a driver of the process [200–202].

In iron metabolism, TFR1 plays a pivotal role by transporting Fe^3+^ into cells, while NCOA4 maintains iron homeostasis through the degradation of ferritin. FPN, which exports Fe^2+^, can induce ferroptosis when inhibited [203, 204].

The regulation of ferroptosis heavily relies on the system xc − /GSH/GPX4 axis, which encompasses cystine-glutamate exchange, GSH synthesis, and the function of GPX4 [205]. Decreased expression of GPX4 and SLC7A11 during ferroptosis renders them reliable markers [206] NADPH levels also serve as indicators of ferroptosis sensitivity [207].

The FSP1/CoQ10 and GCH1/BH4 pathways function as separate mechanisms to inhibit ferroptosis, though additional research is needed to fully understand their roles.qRT-PCR, Western blot, and other assays are commonly used to evaluate these pathways and their components [208, 209]. In summary, studying specific genes, proteins, and transcription factors using these methods offers critical insights into the molecular mechanisms underlying ferroptosis.

#### Detection of reaction products in ferroptosis

As an iron-dependent RCD process triggered by lipid peroxidation, ferroptosis results in significant shifts in key cellular metabolites, including ROS, iron (Fe), and lipid peroxides. This review summarizes detection methods for these key ferroptosis markers.

Intracellular iron accumulation triggers ferroptosis by promoting ROS generation via the Fenton reaction. Various probes and kits are used to detect intracellular iron levels [210]. FerroOrange and Phen Green SK (PGSK) are commonly used to detect Fe2+ using fluorescence microscopy or flow cytometry [211, 212]. Another tool, FeRhoNox-1, binds to Fe2+ and forms a stable orange-red fluorescent complex, enabling the observation of intracellular iron dynamics [213]. The Iron Assay Kit provides a quantitative method for measuring ferrous iron levels in tissues and cells by linking iron concentration to optical density [214]. Lysosomal iron ions can be visualized using LysoTracker Green, with images captured via confocal microscopy [215]. Other probes, such as FIP-1, IP-1, Probe 3, RhoNox-1, SiRhoNox-1, and ICL-1, are designed to identify ferrous ions, whereas CP655, FD1, Sensor 1, FS1, and BOD-NHOH specifically target ferric ions [213].

ROS play a pivotal role in cell signaling and tissue homeostasis and can be detected using specific probes. Intracellular ROS levels are commonly measured with 2′,7′-dichlorodihydrofluorescein diacetate (DCFH-DA), which fluoresces green upon oxidation by ROS [215, 216]. Dihydroethidium (DHE) generates red fluorescence when incorporated into DNA as ethidium oxide [217, 218]. Reactive nitrogen species (RNS), such as peroxynitrite (ONOO−) and NO, also contribute to ferroptosis and can be detected using probes like DAF-FM Diacetate, HKYellow-1, and BTMO-PN [216, 219].

Lipid peroxidation, a key feature of ferroptosis, refers to the oxidative breakdown of lipids, especially PUFAs [220]. The C11-BODIPY assay is commonly used to detect lipid peroxidation in cell membranes [221]. In this assay, the oxidation of C11-BODIPY results in a fluorescence color shift from red to green, indicating lipid peroxide accumulation [222]. Additional approaches include quantifying lipid peroxidation byproducts, such as 4-hydroxynonenal (4-HNE) and malondialdehyde (MDA) [223, 224]. MDA forms a chromophore when reacting with thiobarbituric acid, and its levels can be measured using specialized test kits [225]. Researchers also employ thiobarbituric acid-reactive substances (TBARS) assays to evaluate lipid peroxidation in cellular systems [226].

## Disulfidptosis

### The metabolic mechanism of disulfidptosis

Disulfidptosis is an emerging form of RCD distinct from other established modalities such as apoptosis and ferroptosis [227]. This novel cell death process is characterized by excessive disulfide stress within the cell, primarily resulting from the accumulation of cystine and other disulfide molecules [228]. The key role of SLC7A11, a cystine/glutamate transporter, in regulating both the suppression of ferroptosis and the induction of disulfidptosis highlights the complex interplay and regulatory intricacies of various cell death mechanisms [228, 229].

SLC7A11 plays a crucial role in importing cystine into cells, promoting GSH synthesis, and enhancing GPX4’s ability to protect against ferroptosis [230]. However, in a paradoxical manner, overexpressing SLC7A11 has been found to trigger substantial cell death under glucose-deficient conditions, a phenomenon known as disulfidptosis [228]. This form of cell death results from the buildup of disulfide bonds caused by the excessive reduction of cystine in the cytosol [229].

From a metabolic perspective, cancer cells exhibiting high SLC7A11 expression (SLC7A11-high cells) import large quantities of cystine into their cytosol [231]. Cystine, an amino acid with very low solubility, becomes toxic at elevated concentrations [232]. To mitigate this toxicity, SLC7A11-high cells quickly convert cystine into the more soluble cysteine, a reaction that depends on NADPH as a reducing agent [233]. This process, however, results in substantial NADPH depletion, as NADPH is mainly produced from glucose through the pentose phosphate pathway [234].

As a result, cancer cells with high SLC7A11 expression (SLC7A11-high cells) become highly dependent on glucose to provide the NADPH required for the rapid conversion of cystine to cysteine. When glucose is scarce, these cells experience NADPH depletion, leading to the abnormal accumulation of cystine and other disulfide compounds [229]. This accumulation causes disulfide stress, ultimately triggering rapid disulfide-dependent cell death.

Disulfidptosis is closely linked to metabolic pathways, particularly those involving SLC7A11, NADPH, and the Rac1-WAVE regulatory complex (WRC). To develop precise therapeutic strategies for modulating disulfidptosis, a thorough understanding of the metabolic mechanisms driving this form of cell death is essential [235].

In SLC7A11-high cancer cells, SLC7A11 facilitates cystine import in exchange for glutamate export, a process critical for maintaining intracellular redox balance by promoting GSH synthesis, a key antioxidant [229]. However, under glucose deprivation, these cells experience NADPH depletion, impairing the conversion of cystine to cysteine. This disruption leads to the accumulation of cystine and disulfide stress, which triggers disulfidptosis [236].

NADPH, a crucial cofactor in redox processes, is mainly produced via the pentose phosphate pathway (PPP) when glucose is available. In cells with high SLC7A11 expression, NADPH serves as a reducing agent for converting cystine to cysteine [237]. Under glucose-deprived conditions, NADPH levels decline, impairing the reduction of cystine to cysteine [238]. This leads to the accumulation of cystine and other disulfide compounds, causing disulfide stress and ultimately inducing disulfidptosis.

The Rac1-WRC pathway plays a vital role in controlling actin cytoskeleton dynamics, especially during lamellipodia formation [227, 239]. Rac1, a small GTPase from the Rho family, activates the WRC, leading to the release of the WAVE homology 2 (WH2)-Central-Acidic (WCA) motif, which initiates actin assembly via the Arp2/3 complex [240, 241]. Under disulfide stress, the actin network in lamellipodia becomes susceptible to disulfide bond formation among actin proteins, intensifying the disulfidptotic process.

The activation and function of Rac1 and the WRC are precisely regulated by multiple factors, such as guanine nucleotide exchange factors (GEFs), GTPase-activating proteins (GAPs), guanine nucleotide-dissociation inhibitors (GDIs), and post-translational modifications [242–244]. The equilibrium between Rac1’s active (GTP-bound) and inactive (GDP-bound) states is critical for modulating downstream signaling pathways that control actin polymerization and lamellipodia formation [242, 244]. Likewise, the integrity and activity of the WRC rely on the proper functioning of its components, including WAVE, CYFIP1/2, NCKAP1, HSPC300, and Abi1/2/3 [245].

Beyond the Rac1-WRC pathway, other regulators of actin polymerization, including the Arp2/3 complex, formins, and tandem-monomer-binding (TMB) nucleators, may also play a role in disulfidptosis. The Arp2/3 complex drives the creation of branched actin networks, formins facilitate the elongation of linear actin filaments, and TMB nucleators assemble actin monomers to initiate polymerization [246, 247]. Further research is needed to elucidate the precise contributions of these factors to disulfidptosis and their potential as targets for therapeutic intervention.

**Fig. 3:**
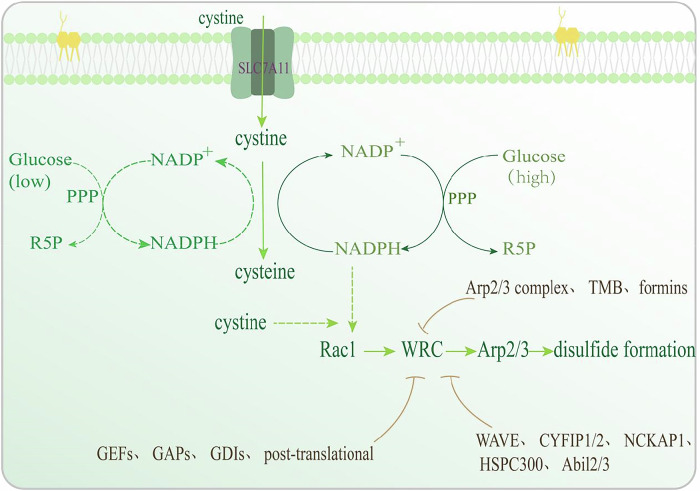
The metabolic mechanism of disulfidptosis. Under glucose-deprived conditions, a decline in NADPH levels, impairing the reduction of cystine to cysteine.Rac1 activates the WRC, leading to the release of the WH2-WCA motif, initiating actin assembly via the Arp2/3 complex.

In summary, the metabolic pathways involved in disulfidptosis are intricate and encompass multiple interconnected components. Deeper insights into these pathways will enhance our understanding of disulfidptosis regulation and support the creation of targeted treatments (Fig. 3).

### Disulfidptosis in bone remodeling

The study in Redox Biology uncovered a pivotal mechanism wherein NFATc1-mediated upregulation of SLC7A11 plays a critical role during osteoclastogenesis. As a master transcription factor in osteoclast differentiation, NFATc1 enhances the transcription of SLC7A11, promoting cystine uptake in osteoclast precursors (pre-OCs). This upregulation increases pre-OCs’ susceptibility to thioredoxin reductase 1 (TXNRD1) inhibitors, such as auranofin (AF) and TXNRD1 inhibitor 1 (TRi-1), which induce disulfidptosis [20]. The findings imply that targeting TXNRD1 to trigger disulfidptosis in pre-osteoclasts could be an effective strategy for treating osteoclast-overactive disorders. This approach allows mature osteoclasts to remain functional, ensuring the maintenance of bone homeostasis. This selective killing mechanism avoids the direct inhibition of mature osteoclasts, which may help maintain bone homeostasis.

### Detection of disulfidptosis

A comprehensive suite of detection methods has been developed to investigate disulfidptosis, providing valuable insights into its underlying mechanisms and potential therapeutic implications. qRT-PCR, Western blot, and IHC are routinely employed to quantify the expression levels of SLC7A11, a critical regulator of disulfidptosis [248–250]. These techniques allow for an in-depth examination of SLC7A11’s involvement in both ferroptosis suppression and disulfidptosis induction. Kit-based assays are also utilized to measure NADPH consumption, which is crucial for maintaining redox homeostasis and driving the progression of disulfidptosis [251, 252]. Immunofluorescence techniques enable the visualization of actin cytoskeletal changes, such as contraction and detachment from the cell membrane, which are characteristic of disulfidptosis [253, 254]. In combination with Western blot and mass spectrometry, these methods facilitate the detection of alterations in disulfide bond levels of cytoskeletal proteins, providing detailed information on the formation of abnormal disulfide bonds that contribute to the cell death process [255, 256]. To assess cell death rates, propidium iodide (PI) single staining and TUNEL staining are employed, although the latter may not be specific to disulfidptosis [257, 258]. In addition to these methods, drug induction experiments using glucose transporter inhibitors, such as BAY-876 and KL-11743, have demonstrated the ability to induce disulfidptosis in SLC7A11-overexpressing cancer cells. These findings highlight potential therapeutic strategies for targeting disulfidptosis in cancer treatment [259, 260]. Collectively, these approaches form a robust toolkit for studying disulfidptosis and advancing the development of targeted therapies.

## PANoptosis

### The metabolic mechanism of disulfidptosis

The concept of PANoptosis, an inflammatory PCD process, has emerged from research exploring the interactions between inflammasomes/pyroptosis, apoptosis, and necroptosis. Traditionally, these pathways were considered distinct and parallel, with minimal overlap [261]. However, mounting evidence reveals extensive crosstalk and cross-regulation among these cell death modalities. Caspase-8, one of the first identified mediators, serves as a key regulator in both apoptosis and necroptosis pathways, underscoring the interconnectedness of these cell death mechanisms [262–264]. Research has elucidated the interactions between pyroptosis and apoptosis, apoptosis and necroptosis, as well as pyroptosis and necroptosis. These findings have significantly advanced the understanding of the mechanisms driving PANoptosis, which is characterized by the activation of the PANoptosome complex [265]. PANoptosis shares key features with necroptosis, apoptosis, and pyroptosis, as reflected in the acronym “P,” “A,” and “N” of the term. Notably, PANoptosis cannot be exclusively defined by any one of these cell death modes [266].

The PANoptosome consists of sensors, effectors, and adapters, each with distinct compositions and mechanisms that respond to various stimuli [267]. Notable PANoptosomes, including those containing ZBP1, AIM2, RIPK1, NLRP12, and NLRC5, have been identified [268]. Their assembly is primarily regulated by interferon signaling, with IRF1 playing a key role, emphasizing the complex interaction between immune responses and cell death pathways [269].

Each PANoptosome exhibits unique features and functions. For example, the ZBP1-PANoptosome, identified during IAV infection, combines ZBP1, NLRP3, and other components to trigger multiple forms of cell death in response to viral challenges [270, 271]. The AIM2-PANoptosome, which detects dsDNA, induces PANoptosis during HSV-1 and F. novicida infections, significantly contributing to innate immunity and inflammation [272, 273]. The RIPK1-PANoptosome mediates Yersinia-induced cell death, while the NLRP12- and NLRC5-PANoptosomes are involved in inflammasome sensing and TLR-dependent assembly, respectively [274].

Caspases, particularly Caspase-1/3/7, Caspase-8, and Caspase-6, are central to executing PANoptosis [275]. These enzymes act as downstream effectors, orchestrating the complex interactions between diverse cell death pathways within the PANoptosome.

The dual role of PANoptosis in diseases remains a critical area of investigation. Although it exacerbates mortality in COVID-19 and aggravates inflammation and organ damage, it also bolsters host defense against infections and influences various cancer types [276]. Modulating PANoptosis holds therapeutic promise, with molecules such as DKK1 and CurE demonstrating potential in animal models [277, 278]. However, additional studies are essential to clarify the relationship between PANoptosis and diseases and to assess the specificity, efficacy, and safety of potential clinical modulators.

### PANoptosis in bone remodeling

Emerging studies have shed light on the involvement of the long noncoding RNA (lncRNA) MIR17HG in controlling PANoptosis, a recently characterized form of PCD, during the differentiation of osteoblasts under inflammatory conditions [279]. Pro-inflammatory mediators like IL1β, IL6, and TNF-α are recognized for their ability to hinder osteoblast differentiation and trigger apoptosis [280]. Through advanced high-throughput sequencing and bioinformatics approaches, MIR17HG was pinpointed as a pivotal lncRNA associated with both inflammation and PANoptosis in osteoblasts [279]. This link was further corroborated through experimental methods such as qRT-PCR and Western blot, solidifying the role of MIR17HG in inflammatory PANoptosis. These insights emphasize the intricate connection between inflammation, PANoptosis, and osteogenic differentiation, suggesting MIR17HG as a potential target for addressing disorders marked by defective bone formation and inflammatory dysregulation. In a complementary study, Zhang et al. examined the role of microRNA-18a-5p (miR-18a) in modulating PANoptosis and osteogenic differentiation within a TNF-α-driven inflammatory setting [281]. Their research revealed that TNF-α downregulates miR-18a expression in MC3T3-E1 osteoblasts. Functional studies demonstrated that miR-18a acts as a negative regulator of hypoxia-inducible factor-1α (HIF1-α) and NLRP3, and that suppressing miR-18a worsens TNF-α-mediated PANoptosis, impairing osteogenic differentiation. On the other hand, overexpressing miR-18a mitigates these detrimental effects. In vivo experiments further supported these results, positioning miR-18a as a promising therapeutic target for anti-inflammatory approaches in managing osteoinflammatory diseases. Collectively, the work of Zhang et al. [281] underscores the pivotal role of miR-18a in coordinating the complex interplay between inflammation, PANoptosis, and osteogenic differentiation.

### Detection of PANoptosis

Detection methods for PANoptosis encompass a range of approaches. Firstly, the examination of cellular morphology at a microscopic level uncovers distinct features such as condensed chromatin, fragmented DNA, cell shrinkage, membrane damage, and the development of apoptotic bodies. Subsequently, pinpointing pivotal proteins linked to RCD is crucial [144, 282, 283]. This is accomplished using methods like qRT-PCR and Western blot to identify molecular markers associated with pyroptosis (e.g., CASP1, GSDMD, GSDME, AIM2, MEFV, NLRP3), apoptosis (e.g., CASP8, CASP3, CASP7, BCL2, BAX), and necroptosis (p-/t-MLKL, p-/t-RIPK1, p-/t-RIPK3, ZBP1)[284, 285] [272, 286–288]. Selecting one to three indicators for each form of PCD guarantees a thorough assessment. Furthermore, detection of cell death is achieved through techniques like the Cell Counting Kit-8/MTT to gauge cell viability and flow cytometry with annexin V-FITC/PI to distinguish between different cell death pathways [289–292]. Early apoptosis is indicated by annexin V-positive cells alone, whereas dual positivity suggests late apoptosis or pyroptosis [293]. Additional staining methods, such as Y-PRO-1/PI, provide additional insights into necrosis and apoptosis/necrosis [294]. Other detection strategies include Annexin V-FITC and PI double staining, terminal deoxynucleotidyl transferase dUTP nick end labeling, JC-1 staining, ELISA for inflammatory cytokine release, immunoblotting, flow cytometry, and the assessment of NLRP3 inflammasome and CASP1 activation, offering a holistic approach to evaluating PANoptosis [295–297].

## The intersection of the four cell death mechanisms in bone remodeling

Emerging evidence demonstrates that the four novel RCD pathways: ferroptosis, cuproptosis, disulfidptosis, and PANoptosis are not entirely independent but rather exhibit significant mechanistic crosstalk in bone remodeling. Key intersecting nodes include p53-mediated regulation [298], GSH depletion, mitochondrial dysfunction, and inflammatory feedback loops. Understanding these complex interactions is crucial for future RCD research and therapeutic development.

### P53 as a master regulator of multiple RCD pathways

Since its discovery in 1979, the tumor suppressor p53 has been recognized as one of the most biologically significant proteins with immense therapeutic potential. Beyond its classical roles in DNA damage response, cell cycle arrest, apoptosis, and senescence, p53 has emerged as a central coordinator of various novel RCD modalities, including ferroptosis, cuproptosis, disulfidptosis, and PANoptosis [299, 300]. This multifaceted regulation positions p53 as a critical molecular nexus integrating multiple cell death pathways.

### P53 in cuproptosis

P53 enhances cellular sensitivity to cuproptosis through dual mechanisms: transcriptional upregulation of FDX1, the essential copper reductase required for lipoylated protein aggregation, and suppression of SLC31A1, the primary copper importer, thereby modulating intracellular copper homeostasis [22, 301].

### P53 in disulfidptosis

The tumor suppressor promotes disulfidptosis by two distinct pathways: inhibiting SLC7A11-mediated cystine uptake to exacerbate disulfide stress, and interacting with NRF2 to alter redox balance in osteoblasts [228, 229, 302].

### P53 in PANoptosis

P53 exerts precise control over PANoptosis through transcriptional activation of ZBP1 and RIPK1, core components of the PANoptosome complex, and modulation of inflammasome sensors (NLRP3, AIM2), thereby linking PANoptosis to bone inflammatory responses [303, 304].

### p53 in Ferroptosis

While not the primary focus, p53 contributes to ferroptosis regulation via SAT1-ALOX15 axis activation and GPX4 suppression [139].

### Shared triggers: GSH depletion, common node: mitochondrial dysfunction, inflammatory crosstalk

**Fig. 4:**
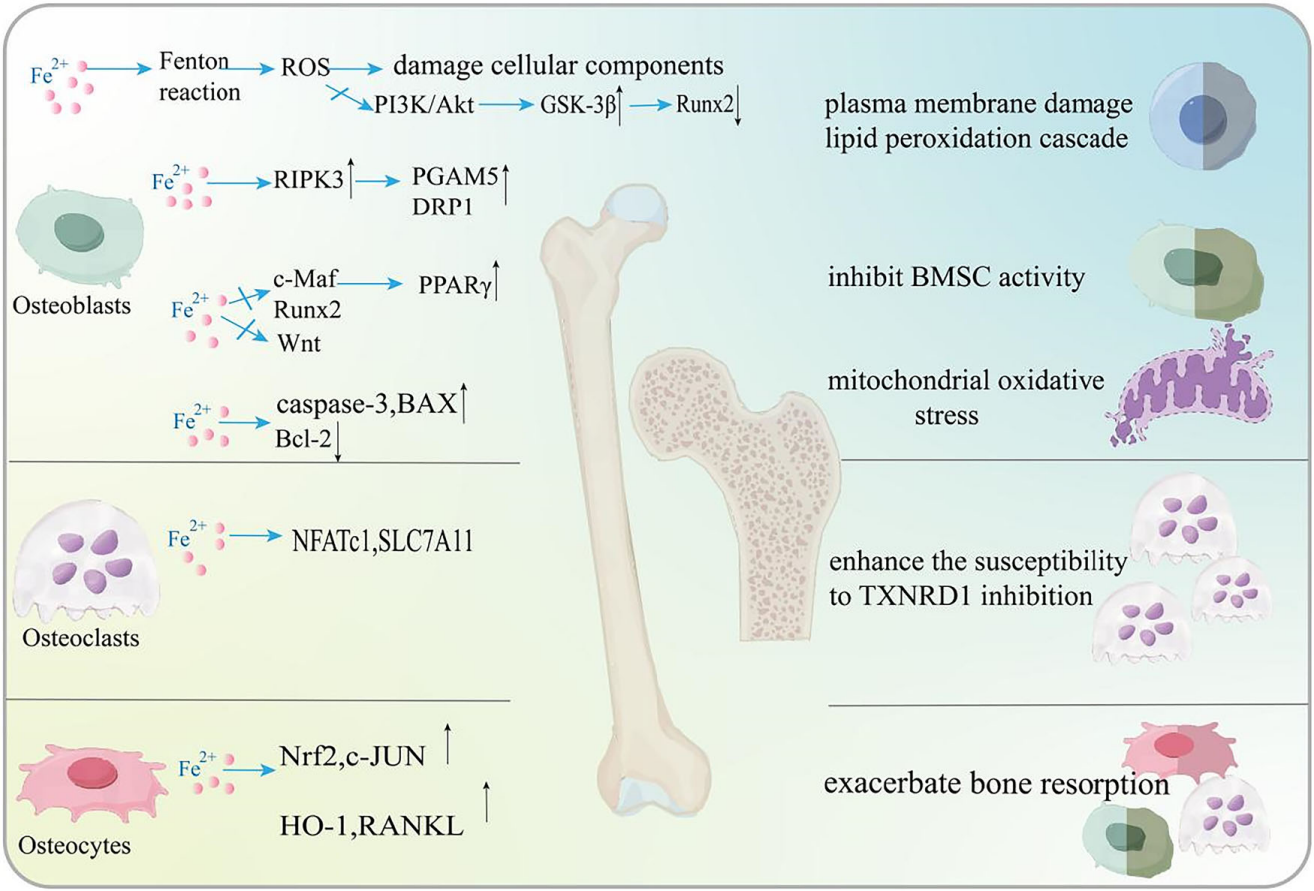
The mechanisms underlying the regulatory effects of RCD on bone remodeling.

Both cuproptosis and ferroptosis require GSH depletion, yet diverge in their subsequent mechanisms:copper-dependent DLAT aggregation versus iron-mediated lipid peroxidation, respectively [22, 305]. Notably, p53/ROS-induced SLC7A11 inhibition disrupts GSH synthesis, simultaneously sensitizing cells to both disulfidptosis and PANoptosis activation [228, 306]. Cuproptosis and ferroptosis both localize to mitochondria (FDX1, ACSL4), where p53-driven metabolic reprogramming (TCA cycle disruption) amplifies oxidative damage [22, 133].PANoptosis-derived DAMPs (HMGB1) create a pro-death microenvironment that exacerbates ferroptosis and cuproptosis in neighboring osteoclasts [272] (Fig. 4).

## Bone remolding-treatment implications on ferroptosis, cuproptosis, disulfidptosis, and PANoptosis

### Natural products targeting ferroptosis for OP

Ferroptosis, a form of cell death marked by iron-driven lipid peroxidation and subsequent membrane disruption, has been linked to OP due to its role in promoting oxidative stress and ROS buildup [307]. Given its strong connection to OP, strategies aimed at inhibiting ferroptosis have gained attention as a potential therapeutic approach to reduce bone loss and improve bone health.

In studies using animal models of OP, treatments aimed at inhibiting ferroptosis have shown encouraging results. For example, in mice with Type 2 Diabetic Osteoporosis (T2DOP), melatonin was found to prevent osteoblast ferroptosis by activating the Nrf2/HO-1 signalling pathway, thereby preserving bone structure and integrity [308]. Similarly, mitochondrial ferritin (FtMt) mitigated ROS accumulation and protected osteoblasts from ferroptosis in T2DOP mice. The glucose-lowering drug metformin inhibited osteoblast ferroptosis, potentially through activation of Runx2/Cbfa1 and AMPK, highlighting its potential in treating diabetic OP [309, 310].

In the context of postmenopausal osteoporosis (PMOP), targeting ferroptosis has also emerged as a potential therapeutic approach. For instance, the HIF-1α inhibitor 2-methoxyestradiol (2ME2) was shown to trigger ferroptosis in osteoclasts and reduce bone loss in OVX mice, a well-established PMOP mode [311]. These results highlight the potential of regulating iron metabolism and ferritinophagy central mechanisms in ferroptosis as a promising strategy for treating PMOP.

In glucocorticoid-induced osteoporosis (GIOP), ferroptosis has been implicated in bone loss and the deterioration of trabecular structure. Studies have shown that endothelial cell-secreted exosomes (EC-Exos) can protect osteoblasts from ferroptosis, preserving bone microstructure in GIOP mice [312]. Furthermore, EC-Exos were found to reduce osteoclast activity and mitigate bone loss in OVX mice, underscoring the therapeutic promise of targeting ferroptosis in OP [313]. In a related study, Zhao et al. revealed that ATF3 promotes high glucose-induced ferroptosis in osteoblasts by inhibiting system Xc- in T2DOP [314].

High glucose levels in individuals with diabetes hinder the ability of BMSCs to differentiate into osteoblasts, playing a significant role in the development of DOP, a disorder marked by weakened bone structure and increased fracture risk [315–317]. To address this, Li and colleagues developed tFNA-Cur, a nanoparticle formulation utilising tetrahedral framework nucleic acid (tFNA) technology. This formulation efficiently delivers curcumin to the bone marrow, significantly enhancing its bioavailability and stability [318].

Both in vitro and in vivo experiments have shown that tFNA-Cur significantly improves mitochondrial function by activating the Nrf2/GPX4 signalling pathway [319]. This dual-action mechanism not only suppresses ferroptosis in BMSCs but also enhances their ability to differentiate into osteoblasts under high-glucose conditions. Consequently, tFNA-Cur promotes bone formation, making it a promising candidate for treating DOP. Its ability to specifically target ferroptosis further highlights its potential as a multifaceted therapeutic agent for a range of conditions.

A recent study found that icariin (ICA) decreased iron accumulation in the bone marrow of mice with OP, although the exact mechanisms were not fully elucidated. In a follow-up study, scientists evaluated the effects of ICA on ferroptosis using a rat model of osteoporotic fractures [320].

In in vitro studies, icariin (ICA) effectively lowered ROS levels and reduced the expression of GPX4 and the cystine-glutamate antiporter (SLC7A11) in osteoblasts treated with erastin, a ferroptosis inducer. Concurrently, ICA activated the Nrf2 signalling pathway and increased the expression of factors related to osteogenesis, highlighting its dual role in inhibiting ferroptosis and stimulating bone formation [320].

These findings were further confirmed using an osteoporotic fracture rat model. In in vivo experiments, ICA was shown to significantly enhance trabecular bone density, promote callus formation, and support the transformation of fibrous callus into bone [320]. Moreover, ICA increased the expression of GPX4, Nrf2, and Runx2 at the fracture site while reducing levels of the pro-apoptotic gene Bax, underscoring its potential as a therapeutic agent for improving bone healing [320].

### Targeting ferroptosis for periodontitis

Periodontitis, a chronic inflammatory condition affecting oral health, is marked by the gradual degradation of tissues supporting the teeth, which can result in tooth loss in severe cases [321, 322]. The development of this disease involves intricate interactions among diverse cell types and molecular mechanisms [323, 324].

#### Osteoclast

Emerging research has identified the presence of ferroptosis in osteogenic cells, such as osteoblasts and osteocytes, during periodontitis. Inflammatory triggers induce ferroptosis, leading to the release of pro-inflammatory cytokines like RANKL and IL-6 from osteocytes. These cytokines play a pivotal role in promoting osteoclastogenesis, the formation and activation of osteoclasts, which are key players in bone resorption [325, 326]. Saikosaponin A (Ssa), a triterpene saponin derived from Bupleurum falcatum, has shown promising effects in reducing osteoclastogenesis and bone loss in periodontitis models. Ssa induces ferroptosis in osteoclasts by promoting lipid peroxidation and mitochondrial damage, which inhibits osteoclast differentiation and function, ultimately leading to reduced bone resorption. The Nrf2/SCL7A11/GPX4 signalling pathway, responsible for regulating the cellular antioxidant response and ferroptosis susceptibility, is involved in Ssa’s mechanism of action [327]. Inhibiting ferroptosis has gained attention as a potential therapeutic approach to manage inflammation and curb bone loss in periodontitis. The work of Tang et al. reinforces this approach, highlighting the efficacy of ferroptosis inhibitors, including liproxstatin-1, as a promising treatment option. In animal models, these inhibitors have proven effective in alleviating bone resorption and inflammation induced by periodontitis, thus validating the therapeutic potential of ferroptosis modulation in this context. [325].

#### Osteoblast

Emerging studies have provided new insights into the intricate interplay between ferroptosis, a type of iron-mediated cell death driven by lipid peroxidation, and the process of osteogenic differentiation. Building on the work of Bao et al., studies have shown that the ferroptosis inducer erastin suppresses osteogenic differentiation and mineralisation in primary osteoblasts, underscoring the detrimental effect of ferroptosis on bone formation. On the other hand, ferroptosis inhibitors such as ferrostatin-1 have been shown to stimulate osteogenesis, highlighting their potential as therapeutic agents for enhancing bone formation [328].

A study exploring the role of ferroptosis in alveolar osteocytes during diabetic periodontitis utilised C57/BL6 male mice with induced disease, treated either with or without resveratrol. Through micro-CT, histology, and IHC, the researchers evaluated the severity of periodontitis and the presence of ferroptosis in alveolar osteocytes. In vitro experiments, MLOY4 osteocytes exposed to P. gingivalis-derived lipopolysaccharide (LPS) and advanced glycosylated end products (AGEs)—mimicking diabetic periodontitis—showed that the condition worsened disease progression, suppressed GPX4 and SLC7A11 expression in alveolar osteocytes, and induced osteocyte ferroptosis. Importantly, resveratrol mitigated these effects, potentially by reactivating the SLC7A11/GPX4 axis and lowering the levels of pro-inflammatory mediators [329]. Furthermore, recent studies have indicated that IL-17 administration confers resistance to erastin-induced osteoblast ferroptosis and promotes osteogenesis [330]. This protective mechanism of IL-17 may be linked to the interaction between phosphorylated signal transducer and activator of transcription 3 (p-STAT3) and nuclear factor erythroid-2-related factor 2 (NRF2)[328]. These findings further underscore the therapeutic potential of modulating ferroptosis pathways, especially through ferroptosis inhibitors like ferrostatin-1 and cytokines like IL-17, to treat conditions associated with osteoblast dysfunction and bone loss, such as periodontitis.

Overall, this body of research emphasizes the intricate relationship between ferroptosis and bone formation. It suggests that diabetic periodontitis induces ferroptosis in both alveolar osteocytes and osteoblasts, and highlights the therapeutic potential of resveratrol and other ferroptosis modulators in reversing these effects and promoting bone health. This opens up promising avenues for future investigations aimed at combating bone diseases linked to ferroptosis. We have listed the therapeutic targets for ferroptosis in osteoporosis and periodontitis in Table 1.

**Table 1:** Therapeutic targets for ferroptosis in osteoporosis and periodontitis.

Drugs	Mechanism for the treatment of osteoporosis	RCD	Drugs	Mechanism for the treatment of periodontitis
tFNA-Cur	A nanoparticle formulation. Activates the Nrf2/GPX4, enhances curcumin bioavailability and stability, and mitochondrial function. Inhibits ferroptosis in BMSCs. Promotes osteogenic differentiation.Stimulates bone formation.	Ferroptosis	Ssa	Promotes E lipid peroxidation and mitochondrial damage. Inhibits osteoclast differentiation and function. Includes the Nrf2/SCL7A11/GPX4, which regulates the cellular antioxidant response and ferroptosis.
FtMt	Mitigates ROS accumulation and protects osteoblasts.	Ferroptosis	Liproxstatin-1	Alleviates bone resorption and inflammation.
Metformin	Inhibits osteoblast ferroptosis. Runx2/Cbfa1 and AMPK activate metformin.	Ferroptosis	Ferrostatin-1	Stimulates osteogenesis.
2ME2	HIF-1α inhibitor. Induces osteoclast ferroptosis and delays bone mass loss.	Ferroptosis	Resveratrol	Restores SLC7A11/GPX4 axis. Reduces pro-inflammatory expression.
EC-Exos	Rescue osteoblasts、protect bone microstructure.	Ferroptosis	IL-17	Involve the interaction of pSTAT3(with nuclear factor NRF2).
ATF3	Suppresses system Xc-.	Ferroptosis		
Melatonin	Activates the Nrf2/HO-1 pathway. Protects bone microarchitecture.	Ferroptosis		
ICA	Reduces iron deposition, ROS, geneBax, and SLC7A1-1 and upregulates the Nrf2, GPX4, Nrf2, and Runx2. Increases trabecular bone density, accelerates callus formation, and facilitates the transition from fibrous to osseous callus.			

### Products targeting cuproptosis

#### Targeting cuproptosis for OP

Recent studies have emphasized the role of cuproptosis, a newly identified type of cell death triggered by excessive copper ions, in the context of OP [331]. Researchers utilised the GSE56815 dataset, analyzing 40 osteoporotic samples to investigate cuproptosis regulators and immune signatures [332]. By applying Weighted Gene Co-expression Network Analysis (WGCNA), they identified distinct clusters of genes related to cuproptosis and patterns of immune infiltration. In their study, Teschke and Eickhoff focused on overlapping differentially expressed genes from osteoporotic samples with cuproptosis-associated genes, uncovering 16 significantly expressed cuproptosis-related genes. They also pinpointed six hub genes and 59 phenotype-associated genes, which were enriched in inflammatory responses and signalling pathways [333]. Immune infiltration analysis revealed two clusters, one of which displayed elevated immune activity. Genes like COX19, MAP2K2, and FDX1 showed strong correlations with immune cell infiltration and were linked to OP prognosis. Additionally, a predictive model incorporating five genes, developed using the random forest algorithm, demonstrated strong performance in external validation. Multiple validation methods confirmed the model’s accuracy in predicting OP subtypes, emphasizing the potential of cuproptosis-related markers for diagnosis, subclassification, and precision medicine [332].

In summary, emerging studies have sparked significant interest in the relationship between cuproptosis and OP, leading to the synthesis of potential links between the two. However, the exact mechanisms of cuproptosis remain unclear, limiting direct clinical applications. Further research is needed to clarify these mechanisms, which could open new clinical avenues. Furthermore, advancements in nanotechnology are exploring nano-formulations that leverage the cuproptosis mechanism to regulate cellular function by modulating intracellular copper levels, offering promising strategies for developing effective treatments for OP.

#### Targeting cuproptosis for periodontitis

Cuproptosis has been implicated in the progression of periodontitis [334]. Studies suggest that macrophages, key players in immune defence and tissue homeostasis, show increased expression of cuproptosis-related markers when exposed to LPS in mouse models of periodontitis [335]. The copper chelator Tetrathiomolybdate (TTM) effectively reduces cuproptosis by promoting autophagosome formation and enhancing mitophagy-related gene expression, while counteracting LPS-induced suppression of autophagic flux. Additionally, TTM mitigates LPS-induced lysosomal dysfunction, including the impaired fusion of autophagosomes with lysosomes, disruption of lysosomal acidic environments, increased lysosomal membrane permeability, and the secretion of cathepsin B. In periodontitis-afflicted mice, TTM treatment decreases cuproptosis, enhances autophagic flux, and lowers cathepsin B levels. These findings suggest that modulating cuproptosis through TTM administration holds promise as a therapeutic strategy for mitigating macrophage dysfunction and intervening in the inflammatory processes of periodontitis.

A recent study explored potential therapeutic agents targeting disulfidptosis in periodontitis by analyzing data from the Gene Expression Omnibus (GEO) database. Six genes—SLC7A11, SLC3A2, RPN1, NCKAP1, LRPPRC, and NDUFS1—were found to be linked to disulfidptosis in periodontitis. Using these findings, researchers developed a ceRNA network, which predicted three promising therapeutic compounds: ME-344, NV-128, and RILUZOLE. These drugs exhibited strong binding affinity to the target genes, indicating their potential as innovative treatments for periodontitis through the regulation of disulfidptosis. Collectively, these studies underscore the therapeutic potential of targeting cuproptosis and disulfidptosis to alleviate macrophage dysfunction and address the inflammatory processes underlying periodontitis. We have listed the therapeutic targets for cuproptosis in OP and periodontitis in Table 2.

**Table 2:** Therapeutic targets for RCD in osteoporosis and periodontitis.

Drugs	Mechanism for the treatment of osteoporosis	RCD	Drugs	Mechanism for the treatment of periodontitis
COX19, MAP-2K2, FDX1	Strongly correlated with immune cell infiltration and associated with osteoporosis prognosis.	Cuproptosis	ME-344, NV-128, RILUZOLE	Demonstrated good affinity for target genes(SLC7A11, SLC3A-2, RPN1, NCKAP1, LRPPRC, NDUFS1).
TXNRD1 inhibitors	Increases bone cystine content, reduces osteoclast numbers, and mitigates bone loss.	disulfidptosis	Target molecules	MLKL, DCN, IL1B, and IL18. Reduce inflammation and tissue destruction.
PGRMC2	Modulates the differentiation of monocytes into macrophages and influences BM-MSC behavior.	disulfidptosis	ZBP1 Inhibitor	Reduces inflammatory cytokine secretion and cell death.
BMDMs	Essential cells in macrophage activation and osteolysis.	PANoptosis	Caspase-1 Inhibitor	Alleviate inflammation and tissue damage.
Apelin-13	Regulates autophagy, apoptosis, and inflammation, exerting multifaceted protective effects on bone.	PANoptosis	RIPK1/3 Blockor	Prevents necroptotic cell death and subsequent inflammation.
		PANoptosis	CEBPG, TFA-P2C, BNIP3	Tissue biomarkers for periodontitis.
		PANoptosis	TTM	Promotes autophagosome formation and enhances mitophagy-related gene expression, while counteracting LPS-induced suppression of autophagic flux. Mitigates LPS-induced lysosomal dysfunction. Increases lysosomal membrane permeability and the secretion of cathepsin B. Enhances autophagic flux and lowers cathepsin B levels.

### Products targeting disulfidptosis

#### Targeting disulfidptosis for OP

Researchers have recently embarked on a novel therapeutic approach for OP, focusing on disulfidptosis and shedding light on the previously underexplored role of its regulators. By analyzing the GSE56815 dataset, diagnostic clusters and immune landscapes associated with disulfidptosis have been uncovered. Through RNA sequencing, seven key regulators were identified: FLNA, ACTB, PRDX1, SLC7A11, NUBPL, OXSM, and RAC1. A random forest model pinpointed FLNA, SLC7A11, NUBPL, and RAC1 as potential risk markers for OP. A validated nomogram model further underscored the clinical significance of these findings. Using consensus clustering, OP samples were divided into two distinct subgroups, with cluster B exhibiting a strong association with monocyte-mediated immunity and osteoclastogenesis. RNA sequencing confirmed the expression patterns of PRDX1 and OXSM, aligning with the predictions from bioinformatics analysis.

In another study conducted by Chengzhen Pan and colleagues, disulfidptosis was found to play a role in the progression of OP, with RPN1 emerging as a potential therapeutic target for kaempferol [336]. By analyzing the differential expression of disulfide-related genes and applying machine learning techniques, RPN1 was identified as a significant risk factor for OP. Further validation across multiple datasets confirmed the aberrant overexpression of RPN1 in patients with OP, which was linked to specific immune alterations and biological processes. A predictive ceRNA network involving RPN1 was constructed, and molecular docking experiments demonstrated a strong binding affinity between kaempferol and RPN1. In vivo experiments in OVX rats revealed that kaempferol improved bone microstructure and reduced the abnormal expression of RPN1, positioning RPN1 as both a diagnostic biomarker and therapeutic target for kaempferol in OP treatment.

A study published in Redox Biology demonstrated that TXNRD1 inhibitors increased cystine levels in bone, decreased osteoclast numbers, and reduced bone loss in OVX mice, providing strong evidence for the therapeutic potential of targeting disulfidptosis in bone-related disorders [20]. Additionally, recent research has identified progesterone receptor membrane component 2 (PGRMC2) as a key player in the development of PMOP through its involvement in disulfidptosis [337]. PGRMC2 was found to be highly expressed in macrophages within bone tissue and prominently localized in bone marrow mesenchymal stem cells (BM-MSCs) from PMOP patients [338]. Reduced PGRMC2 levels were observed in OVX mice, a model for PMOP. Mendelian randomisation analysis further indicated a significant influence of PGRMC2 on OP risk, suggesting its potential protective role against the disease. These findings imply that PGRMC2 may regulate monocyte-to-macrophage differentiation and modulate BM-MSC activity, thereby affecting PMOP progression and severity through its role in disulfidptosis.

#### Targeting disulfidptosis for Periodontitis

Emerging research has highlighted the potential significance of disulfidptosis in the development of periodontitis, connecting oxidative stress and increased disulfide accumulation to the onset of this newly identified cell death mechanism in periodontal tissues. Yixin Fan and colleagues established a connection between disulfidptosis and periodontitis, identifying six genes—SLC7A11, SLC3A2, RPN1, NCKAP1, LRPPRC, and NDUFS1—that are involved in disulfidptosis in this context [339]. Based on their strong binding affinity to these target genes, the study proposed three promising therapeutic candidates—ME-344, NV-128, and RILUZOLE—for the treatment of periodontitis associated with disulfidptosis.

In parallel, Fu et al. [340] conducted bioinformatics analysis combined with experimental validation, identifying 27 disulfidptosis-related transcripts with altered expression in periodontitis. Among them, six were identified as tissue biomarkers for periodontitis, with CEBPG, TFAP2C, and BNIP3 demonstrating the highest classification accuracy.We have lists the therapeutic targets for disulfidptosis in OP and periodontitis in Table 2.

### Products targeting PANoptosis

#### Targeting PANoptosis for OP

While there is no direct evidence linking PANoptosis to OP, a study by Gong et al. [341] published in International Immunopharmacology suggests that PANoptosis could play a significant role in the prevention and treatment of bone diseases, including OP and bone loss. PANoptosis, an inflammatory PCD pathway encompassing apoptosis, necrosis, and pyroptosis, represents a complex form of cell death with significant implications for bone health.

The study highlights PANoptosis’s role in regulating bone marrow-derived macrophages (BMDMs), essential cells in macrophage activation and osteolysis [342, 343]. BMDMs are pivotal in the pathophysiology of bone disorders, including periprosthetic osteolysis (PPO) and osteolysis induced by wear particles from joint replacements [344–346]. By modulating BMDMs, PANoptosis could influence bone homeostasis and the progression of bone diseases.

Furthermore, the study emphasizes the crosstalk between various forms of RCD, including PANoptosis, autophagy, and apoptosis, and their regulation by molecules like Apelin-13. Apelin-13, an adipokine peptide hormone, has been shown to regulate autophagy, apoptosis, and inflammation, exerting multifaceted protective effects on bone [347]. The regulation of these different RCD pathways by Apelin-13 may represent a key mechanism for its bone-protective effects [348]. Considering the essential function of BMDMs in osteoclastogenesis and maintaining bone balance, the study proposes that focusing on PANoptosis could offer a promising approach to alleviate bone-related conditions. Modulating PANoptosis could influence BMDM function and reduce bone loss associated with conditions such as OP.

In summary, although the direct connection between PANoptosis and OP remains to be fully established, the findings presented by Gong et al. provide strong evidence that PANoptosis and related forms of RCD may serve as important targets for future research aimed at preventing and treating bone diseases. The regulation of these processes by molecules like Apelin-13 offers promising new avenues for developing therapeutic strategies focused on bone protection.

#### Targeting PANoptosis for periodontitis

Periodontitis, a persistent inflammatory disorder impacting the periodontal structures, is marked by the degradation of the periodontal ligament, resorption of alveolar bone, and the ultimate loss of teeth [349]. Recent studies have underscored the pivotal involvement of PANoptosis in the progression of this condition. PANoptosis, a complex process encompassing pyroptosis, apoptosis, and necroptosis, opens up novel avenues for therapeutic intervention in periodontitis [350–352].

In 2022, Liu et al. [350] investigated the role of Z-DNA binding protein 1 (ZBP1) in Fusobacterium nucleatum-induced inflammation in apical periodontitis. Their study demonstrated that F. nucleatum infection activates ZBP1, triggering pyroptosis, apoptosis, and necroptosis. Inhibiting ZBP1 reduced inflammatory cytokine secretion and cell death, suggesting that PANoptosis contributes to the disease’s progression. In 2023, Zhang et al. [351] conducted a systematic review of ex vivo and in vivo studies, confirming the presence of pyroptosis, apoptosis, and necroptosis in periodontitis. Elevated levels of key proteins associated with these PCD pathways were observed in both human periodontitis tissue samples and animal models, further supporting the potential occurrence of PANoptosis in periodontitis. More recently, in 2024, Chen et al. [352] used bioinformatics analysis to identify crosstalk pathways and PANoptosis-related genes in periodontitis. Their findings revealed common genes and pathways linking periodontitis with PANoptosis, emphasizing the role of PANoptosis in the shared inflammatory mechanisms of the disease.

Targeting the PANoptosis pathway offers promising therapeutic strategies for periodontitis, given its pivotal role in the disease process. One approach is to inhibit Caspase-1, a key activator of pyroptosis, to alleviate inflammation and tissue damage associated with periodontitis [353]. Another strategy involves blocking the RIPK1/3 signalling pathway, as necroptosis is dependent on this complex; inhibiting this pathway could prevent necroptotic cell death and subsequent inflammation [354]. Additionally, modulating ZBP1, which regulates both pyroptosis and necroptosis, could have widespread effects on multiple aspects of PANoptosis, providing a potential therapeutic intervention. Based on the core genes identified in these studies—such as MLKL, DCN, IL1B, and IL18—therapeutic drugs targeting these molecules could be developed [352]. For instance, drugs that inhibit MLKL oligomerisation or block IL-1β and IL-18 signalling might be tested for their ability to reduce inflammation and tissue destruction in periodontitis [355, 356].

To conclude, accumulating evidence firmly indicates that PANoptosis is a key factor in the development of periodontitis. By targeting this inflammatory PCD pathway, innovative therapeutic strategies could be developed to manage this chronic disease. Additional studies are required to completely unravel the mechanisms behind PANoptosis in periodontitis and to convert these findings into practical medical applications. Targeting PANoptosis holds promise for preventing and treating periodontitis, potentially improving oral health and reducing the burden of associated systemic diseases. We have listed the therapeutic targets for PANoptosis in OP and periodontitis in Table 2.

## Summary and prospects

The mechanisms governing cellular programmed death, a complex physiological and pathological process, are regulated by a variety of factors [357]. In bone remodelling, the roles of osteogenesis and osteoclastogenesis are pivotal, as they collaboratively maintain the dynamic balance of bone formation and resorption [358]. Recent advances have revealed the emergence of novel forms of PCD, such as mitophagy, ferroptosis, cuproptosis, disulfidptosis, and PANoptosis, all of which are intricately linked to the regulation of bone remodelling.

Although research on cuproptosis and disulfidptosis is still in its infancy, studies investigating mitophagy, ferroptosis, and cuproptosis have already extended to basic experiments involving bone cells and animal models. These studies aim to explore their associations with the accumulation of bone morphogenetic proteins (BMPs), osteogenic differentiation, and osteoclastogenesis. In contrast, research on cuproptosis, disulfidptosis, and PANoptosis is primarily bioinformatics-driven, involving gene database screenings, data analyses, and the identification of differential gene expression patterns to establish prognostic markers and models, alongside investigations into their relationship with immune infiltration.

However, significant gaps remain in understanding the mechanisms and pathways through which these novel forms of cell death influence bone remodelling, particularly at the cellular and molecular levels. The absence of fundamental experimental data presents a key opportunity for future research. These forms of PCD operate within a complex interaction network centered on autophagy, encompassing apoptosis, necroptosis, pyroptosis, ferroptosis, cuproptosis, disulfidptosis, and PANoptosis. This intricate network holds considerable potential for the precise, multi-level, and multi-target regulation of bone remodelling processes.

Given the predominance of basic experimental and bioinformatics approaches in research on these novel forms of PCD, there is an urgent need for large-scale, long-term clinical studies to validate these findings. Future research should focus on elucidating the signalling pathways associated with these forms of PCD, investigating the signal transduction mechanisms linked to osteogenic or osteoclastogenic effects of BMPs and other factors in bone remodelling, and designing clinical trials to enable the conversion of these discoveries into therapeutic use. Furthermore, pinpointing pivotal targets within natural plant extracts capable of suppressing these modes of PCD could offer vital clues for crafting innovative therapeutic approaches to treat bone remodelling disorders.

The development of therapeutics targeting RCD pathways faces several critical challenges that must be addressed. A primary obstacle lies in achieving targeted drug specificity, particularly since key regulators of ferroptosis and cuproptosis (including GPX4 and FDX1) exhibit ubiquitous tissue expression patterns. This widespread distribution significantly compromises target selectivity. Furthermore, emerging preclinical evidence reveals an intricate crosstalk between different cell death modalities, where inhibition of specific RCD pathways (notably cuproptosis) can paradoxically trigger compensatory PANoptosis activation, ultimately contributing to therapeutic resistance [359]. The challenge is further compounded by delivery limitations, as current small-molecule inhibitors (such as liproxstatin-1) demonstrate minimal bone tissue specificity with systemic delivery efficiencies remaining below 2% [360]. Within the Chinese research context, additional complexities arise from the inherent tension between the polypharmacological nature of traditional Chinese medicine compounds and the precise mechanistic targeting required for effective RCD modulation.

Moving forward, strategic development should prioritise bone-targeted nanocarrier systems, exemplified by tetracycline-conjugated siRNA liposomes designed for osteoclast-specific delivery. Equally important is the development of combinatorial approaches that simultaneously target multiple cell death pathways, including the design of dual-acting inhibitors against both ferroptosis and cuproptosis mechanisms. The rich pharmacopoeia of Chinese herbal medicine presents unique opportunities to transform these challenges into advantages through systematic high-throughput screening and subsequent optimisation of lead compounds like polydatin derivatives for disulfidptosis modulation. Parallel advances in biomaterial science, particularly through the development of bioinspired mineralised hydrogels and 3D-printed scaffolds replicating trabecular bone architecture, offer promising platforms for localized RCD modulator delivery and represent fruitful avenues for interdisciplinary collaboration.

Several questions remain unanswered, such as whether the extent of cellular programmed death varies between the initial and subsequent phases of bone remodelling, whether there are undetected components of PCD involved in bone remodelling, and how other potential targets might interact with these pathways to influence bone remodelling. Additionally, further exploration is needed into the specific roles of these novel forms of cell death in osteoblasts and osteoclasts, as well as their potential interactions in regulating bone homeostasis. Advancing our understanding of the mechanisms underlying PCD in bone remodelling will be instrumental in developing innovative therapeutic approaches for the treatment of bone-related disorders.
